# Integrated microRNA and mRNA Expression Profiling Identifies Novel Targets and Networks Associated with Ebstein’s Anomaly

**DOI:** 10.3390/cells10051066

**Published:** 2021-04-30

**Authors:** Masood Abu-Halima, Viktoria Wagner, Lea Simone Becker, Basim M. Ayesh, Mohammed Abd El-Rahman, Ulrike Fischer, Eckart Meese, Hashim Abdul-Khaliq

**Affiliations:** 1 Institute of Human Genetics, Saarland University, 66421 Homburg, Germany; viktoria.wagner@ccb.uni-saarland.de (V.W.); lea.becker@uni-saarland.de (L.S.B.); ulrike.fischer@uks.eu (U.F.); eckart.meese@uks.eu (E.M.); 2 Department of Pediatric Cardiology, Saarland University Medical Center, 66421 Homburg, Germany; Mohamed.Abd-El-Rahman@uniklinikum-saarland.de (M.A.E.-R.); Hashim.Abdul-Khaliq@uniklinikum-saarland.de (H.A.-K.); 3 Center for Clinical Bioinformatics, Saarland University, 66123 Saarbrücken, Germany; 4 Department of Laboratory Medical Sciences, Alaqsa University, Gaza 4051, Palestine; bm.ayesh@alaqsa.edu.ps

**Keywords:** microRNA, mRNA, integration analysis, congenital heart defect, Ebstein’s anomaly

## Abstract

Little is known about abundance level changes of circulating microRNAs (miRNAs) and messenger RNAs (mRNA) in patients with Ebstein’s anomaly (EA). Here, we performed an integrated analysis to identify the differentially abundant miRNAs and mRNA targets and to identify the potential therapeutic targets that might be involved in the mechanisms underlying EA. A large panel of human miRNA and mRNA microarrays were conducted to determine the genome-wide expression profiles in the blood of 16 EA patients and 16 age and gender-matched healthy control volunteers (HVs). Differential abundance level of single miRNA and mRNA was validated by Real-Time quantitative PCR (RT-qPCR). Enrichment analyses of altered miRNA and mRNA abundance levels were identified using bioinformatics tools. Altered miRNA and mRNA abundance levels were observed between EA patients and HVs. Among the deregulated miRNAs and mRNAs, 76 miRNAs (49 lower abundance and 27 higher abundance, fold-change of ≥2) and 29 mRNAs (25 higher abundance and 4 lower abundance, fold-change of ≥1.5) were identified in EA patients compared to HVs. Bioinformatics analysis identified 37 pairs of putative miRNA-mRNA interactions. The majority of the correlations were detected between the lower abundance level of miRNA and higher abundance level of mRNA, except for let-7b-5p, which showed a higher abundance level and their target gene, *SCRN3,* showed a lower abundance level. Pathway enrichment analysis of the deregulated mRNAs identified 35 significant pathways that are mostly involved in signal transduction and cellular interaction pathways. Our findings provide new insights into a potential molecular biomarker(s) for the EA that may guide the development of novel targeting therapies.

## 1. Introduction

Ebstein’s anomaly (EA) of the tricuspid valve is a rare congenital heart disease (CHD) with an average prevalence of ~1 per 200,000 live births, accounting for ~1% of all birth defects [1]. It is characterized by an abnormal formation of the tricuspid valve and right ventricular myopathy leading to structural and functional abnormalities [2]. Approximately 50% of EA patients present with other structural cardiac abnormalities like ventricular septal defect (VSD), atrial septal defects (ASD), pulmonary stenosis or atresia (PS/PA), and conduction system abnormalities [2,3,4,5]. Besides, the possible presence of non-compaction, cardiomyopathy may determine the clinical time course and physical performance of the patients. Thus, the manifestation of tricuspid anomaly depends largely on the severity of the defects and other associated cardiac abnormalities and is associated with the clinical course of patients with EA. Therefore, surgical repair or replacement of the tricuspid valve is the most commonly performed cardiac operation to restore normal blood flow and preserve the function of the heart muscle.

EA occurs mostly sporadically, and familial transmission has been rarely observed [6]. It is a genetically heterogeneous disease, in which microdeletions in 1p36 and 8p23.1, and a missense mutation in the actin-binding protein Filamin A (*FLNA*), have been described [5,6,7,8]. Reproductive and environmental factors have also been suggested to play a role in the development of EA [5,6,8]. Although the exact mechanism of CHDs development is still largely unknown, several protein-coding genes have been identified to play a restricted functional role in cardiac development. These genes were found to functionally interact with their intronic microRNAs (miRNAs) in a specific pattern [9,10]. miRNAs are a class of genome-encoded small RNAs (18–22 nucleotides) that serve as important regulators of gene expression via a sequence-specific interaction with the 3′ untranslated region (3′UTR) of the messenger RNA (mRNA), thus decreasing the stability or inhibiting the translation of mRNA [11,12]. Currently, there are 2300 “real” miRNAs in the miRNA database based on miRBase.org [13]. It is assumed, that almost every cellular and biological process is regulated by miRNAs, including processes regulating CHDs [14,15,16,17,18,19,20,21].

In patients with EA, however, investigation of miRNA and mRNA abundance levels is still lacking. Thus, it is conceivable that, in addition to an entire transcriptome and miRNome profiling, the search for a miRNA whose abundance level inversely correlates with the abundance level of mRNA target may demonstrate another layer of the molecular diversity of EA and may potentially be a useful diagnostic and prognostic tool for therapy. Therefore, in this study, we investigated the differences in miRNA and mRNA abundance levels in patients with EA and age and gender-matched healthy volunteers (HVs). We furthermore performed an integrated analysis to identify the mRNA targets of the deregulated miRNAs and to identify the potential pathway(s) that might be involved in the mechanisms underlying EA.

## 2. Materials and Methods

### 2.1. Study Population and Sample Collection

The study population comprised 16 EA cases and 16 age- and gender-matched healthy volunteer controls (HVs). EA patients were diagnosed according to the Carpentier criteria of Ebstein’s Anomaly [22]. A total of 16 patients (10 females and 6 males) were prospectively recruited during the routine cardiac follow-up at the pediatric department of the University Hospital of Saarland. Two-dimensional echocardiography was used to evaluate the anatomy and function of atrioventricular valves and ventricular function in the right and left parts of the heart. Additional cardiac anomalies, such as ASD, VSD, anatomy, and function of the aortic and pulmonary valve were also examined and documented. The diameter of the ascending aorta was used to determine the patients’ Z-score. Moreover, the Left Ventricular end-diastolic Diameter (LVEDD) and the presence of Mitral Valve Prolapse (MVP) were assessed by echocardiography. All HVs underwent clinical examination to rule out any confounding cardiac and extracardiac abnormalities, including measurement of blood pressure, transcutaneous oxygen saturation, and two-dimensional echocardiography. At the time of enrolment, none of the controls took any medication or had elevated blood pressure. Additionally, none of them had any heart abnormality on the echocardiogram.

About 2.5 mL of venous blood was collected from each patient and HV control from the cubital vein in PAXgene blood tubes (BD Biosciences, San Jose, CA, USA). The PAXgene blood tubes were stored at room temperature for 2 h to ensure complete lysis of the blood cells before they were stored at −20 °C until RNA, including miRNA isolation.

The study was approved by the locally appointed Ethics committee of the Ärztekammer des Saarlandes (State Chamber of Physicians of the German federal state of Saarland) (Institutional Review Board (No. 07/18)), in accordance with the Declaration of Helsinki. All patients, their parents, or legal guardians provided their written informed consent.

### 2.2. Conventional Echocardiography for Assessment of LV Function

All patients underwent conventional echocardiography using GE M4S or GE M5S-D Phased Array Ultrasound Transducer with a Vivid E9 Ultrasound System (General Electrics, Fairfield, CT, USA). Patients were examined in the left lateral decubitus position. Initially, conventional echocardiography was performed in accordance with the European Society of Echocardiography recommendations [23]. Pulsed Doppler flow was assessed with simultaneous electrocardiograph recordings in each patient. The usual size of the pulsed Doppler gate was 1.5 mm and the filter was adjusted to 100 Hz. For optimal acquisition, care was taken to direct the transducer beam as close as possible to the Doppler beam. No angle correction was made for the Doppler examination. The mitral valve inflow velocity profiles were recorded with the Doppler sample placed at the tip of the mitral valve in the four-chamber view. Mitral inflow peak velocities were recorded at early (E peak velocity, cm/s) and late (A peak velocity, cm/s) diastole. The ratio of E/A was calculated. In an apical five-chamber view, flow velocity was recorded in the left ventricular outflow tract with the sample volume placed just below the aortic valve. In parasternal short-axis view, flow velocity was recorded in the right ventricular outflow tract with the sample volume placed just below the pulmonary valve. All Doppler-derived parameters were measured offline. The mean value of each parameter was calculated in three consecutive cardiac cycles. The Tei index and the isovolumetric contraction (ICT) and relaxation (IVRT) time were calculated as previously described [24].

### 2.3. RNA Extraction and Quality Assessments

The total RNAs, including miRNAs, were extracted from the blood samples collected in PAXgene™ blood tubes (Becton–Dickinson, Heidelberg, Germany) shortly after echocardiographic evaluation. Extraction was carried out using PAXgene™ Blood miRNA Kit on the QIAcube™ robot (Qiagen, Hilden, Germany), following the manufacturer’s recommendations. DNase I treatment was included to eliminate the residual genomic DNA (Qiagen). The RNA yield and purity were determined using Agilent 2100 Bioanalyzer (Agilent Technologies, Santa Clara, CA, USA) and NanoDrop 2000 Spectrophotometer (Thermo Fisher Scientific, Waltham, MA, USA). All RNA samples fulfilled the quality requirement with an RNA integrity number of ≥7.5.

### 2.4. Analysis of miRNAs and mRNAs by Microarray

The abundance level of miRNAs was performed using the SurePrint™ 8 × 60 K Human v21 miRNA microarrays (Agilent Technologies), according to the manufacturer’s instructions with slight modification. Briefly, 125 ng RNA was labeled, hybridized to the miRNA microarray chip, washed, and the images were acquired using an Agilent DNA microarray scanner (Agilent Technologies). Similarly, the abundance level of mRNAs was performed using the same samples by hybridization onto SurePrint G3 Human Gene Expression v3 8 × 60K microarrays, containing 50,599 biological features (Agilent Technologies), according to the manufacturer’s instructions with slight modification. Briefly, 125 ng total RNA was reverse transcribed, amplified, labeled with cyanine-3 (Cy3), and subsequently hybridized to the mRNA microarray chip. Arrays were washed, and images were acquired using an Agilent DNA microarray scanner (Agilent Technologies). Finally, the Feature Extraction Software (Agilent Technologies) was used to extract miRNA and mRNA expression data.

### 2.5. Microarray Data Analysis

For the miRNA and mRNA microarray evaluation, samples were processed as previously described [25]. Based on the computed sample detection matrix, features were filtered to exclude mRNAs and miRNAs with a detection rate less than 100% in each group (EA and HVs). Obtained expression signals were quantile-normalized and log2-transformed. All samples were preprocessed using R v3.5.1. The further downstream analysis was performed using R with the offside v0.7.6 package for differential expression analysis. Fold-changes based on the log_2_ normalized expression values were calculated. The R stats implementation was used to perform an unpaired two-tailed t-test and *p*-values were corrected using the False Discovery Rate (FDR)-controlling procedure by Benjamini–Hochberg. Adjusted *p*-values smaller than 0.05 were considered significant. To generate all plots shown, either base R functionality was used and/or functions from the ggplot2 v3.2.1, pheatmap v1.0.12, rcolorbrewer v1.1.2, igraph v1.2.6, viridis v0.5.1, and grepel v0.8.1. The packages data.tablev1.12.8, openxlsx v4.1.4, scales v1.1.0, stringr v1.4.0, and rfast v1.9.5 were used to implement common data manipulation tasks. Analysis of potential mRNA-miRNA interactions was performed using R with data.table v1.12.0, corrplot v0.84, and viridisLite v0.3.0. The Spearman correlation coefficient of expression was calculated for each miRNA-mRNA pair for the EA and control group, as well as *p*-value for this correlation. Differences between the two groups were calculated as well. The correlation analysis was performed for all significantly differently expressed miRNAs and mRNAs (*p* < 0.05).

### 2.6. Reverse Transcription and Quantitative Real-Time PCR (RT-qPCR) of miRNA

The abundance level of single miRNA and mRNA was quantified by RT-qPCR using the Biomark HD System (Fluidigm Corporation, CA, USA). The TaqMan™ MicroRNA-Assays (Thermo Fisher Scientific) and Fluidigm^®^ DELTAgene™ Assays (Fluidigm Corporation) were respectively used for miRNA and mRNA, according to the manufacturer’s recommendations. As for the miRNA analysis, the complementary DNA (cDNA) was generated in 12 µL reactions by reverse transcription of 75 ng total RNA using the TaqMan™ MicroRNA Reverse Transcription Kit (Thermo Fisher Scientific), according to the manufacturer’s instructions. Following reverse transcription, 2.5 μL of the generated cDNA was pre-amplified by mixing 12.5 µL of TaqMan™ PreAmp Master Mix (2X) and 3.75 μL of the previously prepared PreAmp Primers Pool (0.2X) in 25 µL reaction volume. As for the mRNA analysis, the cDNA was generated in 5 µL reactions by reverse transcription of 75 ng total RNA using the Reverse Transcription Master Mix (Fluidigm Corporation), according to the manufacturer’s instructions. Following reverse transcription, 1.5 μL of the generated cDNA was pre-amplified by mixing 1.0 µL of PreAmp Master Mix (Fluidigm Corporation) and 0.5 μL of the Pooled Delta Gene assay mix (500 nM, Fluidigm Corporation) in 5 µL reaction volume. A clean-up step with Exonuclease I (New England Biolabs, United Kingdom) was carried out as indicated in Fluidigm’s protocol PN 100-5875 C1. Following the pre-amplification of the cDNA from both miRNA and mRNA, RT-qPCR was carried out with 96.96 Dynamic Array™ IFC for Gene Expression arrays (Fluidigm Corporation) as indicated in Fluidigm’s protocol for miRNA (PN 68000130 E1) and mRNA (PN 100-9792 B1). After loading, the array was placed in the Biomark HD instrument for quantification and detection using a specific thermal cycling protocol for either miRNA or mRNA analysis. RNAU6B and GAPDH were used as reference endogenous controls for miRNA and mRNA, respectively, as previously recommended for this type of sample [14,15,16,17,18,19,21,26]. The data were analyzed with Real-Time PCR Analysis Software (Fluidigm Corporation), according to Fluidigm’s recommendation. Besides, No -Template Control (NTC) and RT-negative control were included in each run.

### 2.7. Overrepresentation and Pathway Analysis

To investigate the significant function of the differentially abundant target genes identified in patients with EA compared to HVs (Supplementary Table S3), the Protein ANalysis THrough Evolutionary Relationships (PANTHER) Classification System was used to classify the target genes according to Gene Ontology terms and KEGG (Kyoto Encyclopedia of Genes and Genomes) pathway annotations [27]. The network of the miRNAs which have a binding site within the 3′UTR target mRNAs was generated by using miRTargetLink software (https://ccb-web.cs.uni-saarland.de/mirtargetlink access date on 28 April 2021) [28].

### 2.8. Statistical Analysis

The results of RT-qPCR experiments are expressed as the median ± standard deviations from triplicates experiments. Comparisons between EA patients and HVs were calculated using an unpaired two-tailed *t*-test in R stats for each miRNA and mRNA. The FDR-controlling procedure by Benjamini–Hochberg was used to correct *p*-values.

## 3. Results

### 3.1. Characteristics of the Study Population

**Figure 1:**
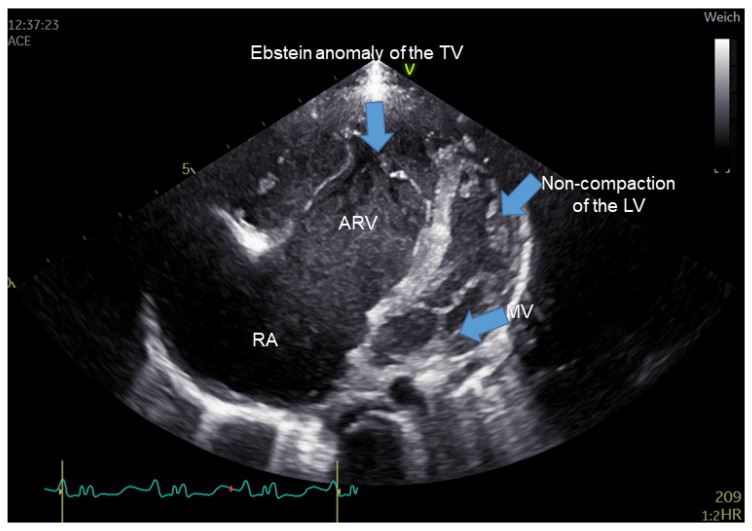
Transthoracic echocardiographic image showing the four-chamber view. The characteristic malformation of severe Ebstein’s anomaly of tricuspid valve in the right heart is clearly seen with an apical displacement of the septal leaflet of the tricuspid valve, leading to marked enlargement of the right atrium and atrialized right ventricle (ARV). The left ventricle shows prominent apical trabeculation suggestive of non-compaction of the left ventricle. ARV, atrialized portion of the right ventricle (ARV); TV, Tricuspid valve; MV, mitral valve; LV, left ventricle; RA: right atrium.

Clinical characteristics of EA patients are displayed in Supplementary Table S1. The mean age of EA patients was 25.8 ± 12.4 years; the mean weight was 63.7 ± 14 kg, and the mean height was 165 ± 8.12 cm. Following the Carpentier criteria, the severity of EA could be classified as mild in 5/16 patients (31.25%), moderate in 9/16 patients (56.25%), and severe in 2 patients (12.50%). Figure 1 shows a representative 2-dimensional echocardiographic result for EA patients. One patient (1/16, 6.25%) underwent reconstructive surgery for the tricuspid valve. Four patients originated from one family with different degrees of EA (25.0%).

The patients could be classified based on the New York Heart Association (NYHA) Functional Classification for heart failure as follows: eight patients (50.0%) were in class I, three patients (18.75%) in class II, and five patients (31.25%) were in class III. Eight patients (50.0%) had compaction cardiomyopathy of the left ventricle and seven patients (43.75%) had associated Supraventricular arrhythmias. The mean systolic blood pressure (SBP) was 120 ± 9.69 mm Hg, the mean diastolic blood pressure (DBP) 67.4 ± 9.32 mm Hg. In seven patients (43.75%) an ASD was detected, two of them had a right-left shunt across the ASD. These two patients had an oxygen saturation of 85% and 93%. The mean LV fractional shortening (LVFS) among patients was 33.9 ± 7.36%, mean LV Tei index was 0.36 ± 0.16, mean ICT 47.69 ± 25.70 ms, mean isovolumic relaxation time (IVRT) 62.46 ± 40.86 ms, mean E/A ratio 1.65 ± 0.80, mean tricuspid annular plane systolic excursion (TAPSE) of 20.6 ± 5.21 mm, mean velocity-time integral across the aortic valve (AVTI) was 26.5 ± 4.99 cm, and mean velocity-time integral across the pulmonary valve (PVTI) was 23.6 ± 5.42 cm.

### 3.2. Correlation Analysis of miRNA and mRNA between EA Patients and Controls

**Figure 2:**
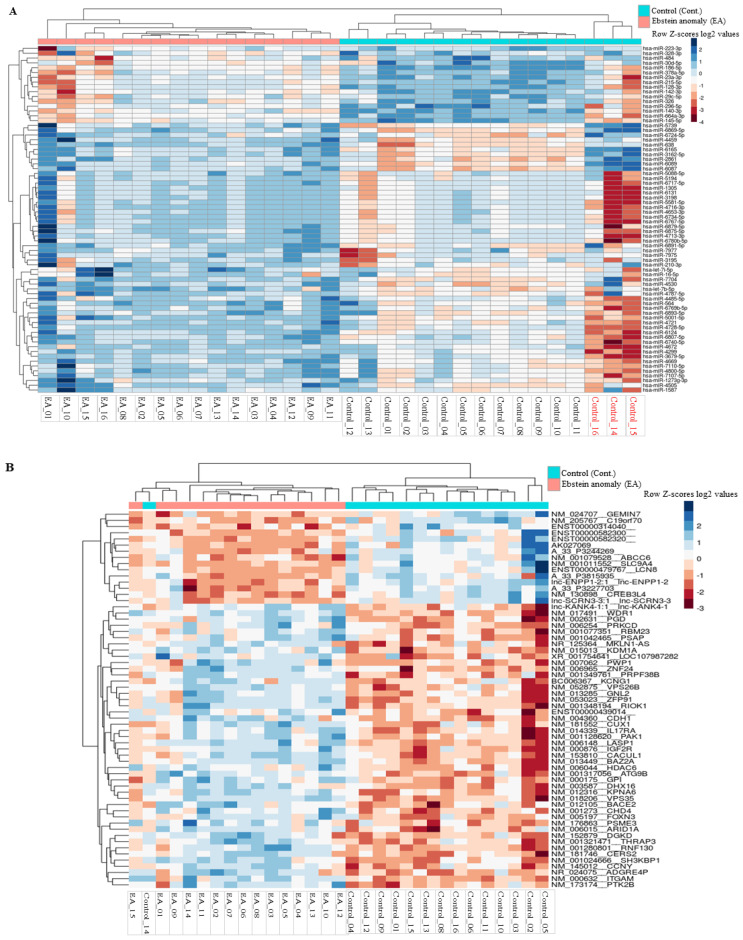
Unsupervised hierarchical clustering (Euclidian distance, complete linkage) of the patients with Ebstein’s Anomaly and Healthy controls based on the (**A**) differentially abundant miRNAs with significant highest variance out of 2549 miRNAs, (**B**) differentially abundant transcripts with significant highest variance out of 50,599 biological features. The heatmap shows miRNAs and transcripts with higher abundance levels in blue, miRNAs with lower abundance levels in red. The light blue and light red lines indicate the two clusters of samples, i.e., Ebstein’s Anomaly and Healthy controls.

As an initial analysis, hierarchical clustering of miRNAs, mRNAs, and samples, i.e., EA patients *versus* HVs based on average linkage and Euclidian distance of the significantly deregulated miRNAs and biological features, was carried out. As for the miRNA profiling, hierarchical clustering of the significantly deregulated miRNA showed that EA patients and HVs were grouped into two distinct clusters with a cluster containing only patients and a second cluster containing only HVs, except for three control samples that were clustered together and showed different expression patterns. These three samples, i. e., control 14, control 15, and control 16, were subsequently excluded from the miRNA array analysis because they showed a strong technical bias. Moreover, the heatmap revealed that some miRNAs were differentially abundant only in the EA patients’ group and/or abundant at a low level in the HVs groups, and vice-versa (Figure 2A). As for the mRNAs profiling, the same hierarchical clustering with the Euclidian distance measure was carried out to measure how the EA patients and HVs relate to each other based on the mRNA abundance levels. For this task, we used the significantly and differentially abundant transcripts with the highest abundant variances out of the 50,599 biological features. As illustrated in Figure 2B, the resulting heatmap of the hierarchical clustering shows a cluster containing mostly EA patients and a second cluster containing most of the HVs. A more detailed distinction between the patients and control groups based on the hierarchical clustering was not possible. Additionally, the heatmap revealed that some differentially abundant transcripts were differentially abundant only in the EA patients’ group and/or abundant at a low level in the HVs groups, and vice-versa (Figure 2B). Moreover, the heatmap shows that there are more transcripts of higher abundance levels in EA than lower abundance levels.

### 3.3. Differential Abundance Analysis between EA Patients and Controls

Differential abundance analyses were performed to identify miRNAs and transcripts that showed either higher abundance level or lower abundance level in the blood samples collected from patients with EA compared to that of HVs. Following background correction, quantile normalization, and log2 transformation, the abundance levels for each group (i.e., EA patients and/or HVs) were identified. By applying an unpaired two-tailed t-test for the miRNA and transcripts that showed a significant change in their abundance level in the considered groups, we found 181 miRNAs and 65 transcripts with significant differences in the patients’ groups compared to that in the HVs group (Supplementary Table S2 and S3, adjusted *p* < 0.05). Considering only the protein-coding genes and removing different transcript variants, 51 transcripts out of 65 transcripts were identified (Supplementary Table S3, adjusted *p* < 0.05). Among the deregulated miRNAs, 49 miRNAs showed significantly lower abundance levels with a fold-change of ≤ 2 and 27 miRNAs showed significantly higher abundance levels with a fold-change of ≥ 2 (Table 1, adjusted *p* < 0.05). Similarly, among the deregulated transcripts, 25 transcripts showed significantly higher abundance levels with a fold-change of ≥1.5, and only 4 transcripts showed significantly lower abundance levels with a fold-change of ≥0.5 (Table 2, adjusted *p* < 0.05). Additionally, our expression profiling analyses did not show statistically significant differences in the miRNAs and mRNAs expression levels related to sex. Likewise, we did not find differences related to age.

### 3.4. Differential Abundance Analysis between EA Patients and Controls

Differential abundance analyses were further carried out to identify miRNAs and transcripts that showed either higher abundance level or lower abundance level in patients with familial Ebstein’s anomaly compared to that in the non-familial Ebstein’s anomaly cases. As for the miRNA analysis, 8 significantly altered miRNAs were identified when comparing the familial (*n* = 4) and non-familial (*n* = 12) cases (Table 3, adjusted *p* < 0.05). Most of these miRNAs showed an altered abundance level in all EA patients as compared to HVs (Table 1, adjusted *p* < 0.05). We next analyzed each group separately, i.e., familial EA cases and non-familial cases. We found, for example, hsa-miR-6085 with higher deregulated abundance level in EA patients with no familial background. As for the mRNA analysis, we did not find mRNAs with significantly different abundance levels between the familial (*n* = 4) and non-familial (*n* = 12) EA cases. The influence of the EA severity could not be analyzed, due to the limited sample size (Family members with severe EA: *n* = 2, Supplementary Table S1).

### 3.5. Validation of Candidate miRNAs and mRNA Targets by RT-qPCR

**Figure 3:**
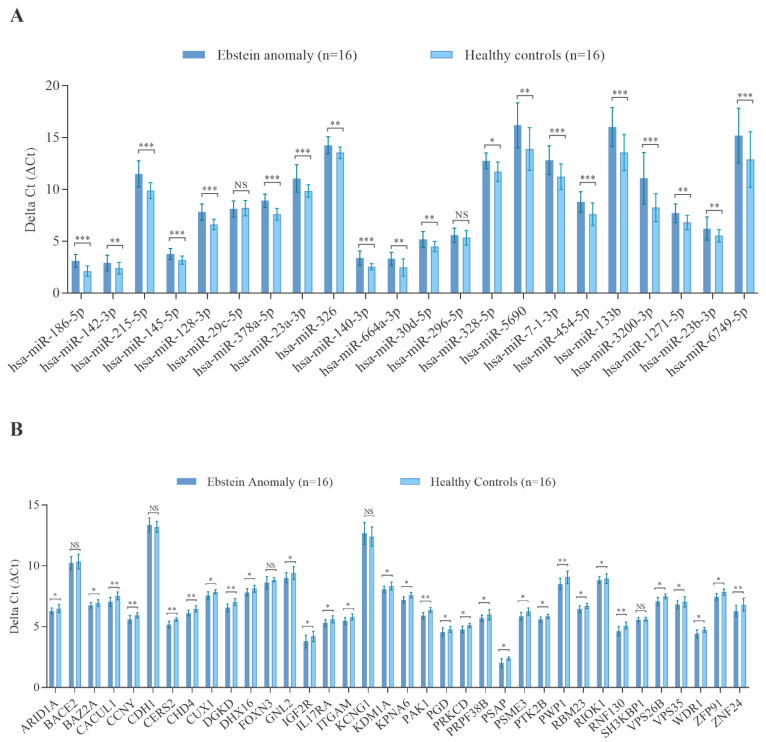
Validated miRNAs and target mRNAs in the blood of patients with Ebstein’s anomaly compared to healthy controls as determined by RT-qPCR. (**A**) Differentially abundant miRNAs, (**B**) differentially abundant mRNAs. Median ΔCt of all Ebstein’s anomaly and ΔCt of all healthy controls (lower ΔCt, higher abundance level). RNAU6B and GAPDH were used as endogenous controls for normalization for miRNA and mRNA, respectively. Un-paired-two-tailed *t*-tests and mean ± standard deviation (STDV) were used to evaluate differences in abundance levels. * *p* ≤ 0.05; ** *p* ≤ 0.01; *** *p* ≤ 0.001.

Using RT-qPCR, the validation of microarray data was performed to re-examine the abundance level of 22 miRNAs and 36 target genes using the same samples, which have been used for the microarray experiments for both miRNA and mRNA profiling. These 22 miRNAs and 36 target genes (as illustrated in Figure 3A,B) were selected based on their differential abundance level in EA patients compared to that in HVs. As shown in Figure 3A, RT-qPCR showed the same direction of abundance changes as the microarray analysis for 21 miRNAs, when comparing the samples from EA patients to HVs. Significant changes in abundance level were confirmed for 20 miRNAs with lower abundance levels (adjusted *p* < 0.05). Similarly, RT-qPCR showed the same direction of abundance changes as the microarray analysis for 34 target mRNAs when comparing the samples from EA patients to HVs (Figure 3B). Significant changes in abundance level were confirmed for 32 target mRNAs with higher abundance levels (adjusted *p* < 0.05).

### 3.6. Integrative Analysis Identified miRNA-mRNA Interaction Network for EA

To further understand the relationship between the identified miRNA and mRNA target changes, and to specifically identify the potentially relevant miRNA–mRNA target interactions, we calculated the correlation coefficient for every miRNA and mRNA target that was significantly deregulated between the EA and HVs, separately for each group. By considering (1) miRNA and mRNA with a fold change of ≥2.0 and ≥1.5, respectively, (2) an inverse correlation, i.e., lower abundance level of miRNA and higher abundance level of mRNA target and vice-versa, (3) miRNAs have binding sites within the 3′UTR region of the identified target genes, and (4) pairs with a difference higher than 0.5 in correlation coefficient between the EA and HVs. A total of 37 interactions were identified, as shown in Supplementary Table S4.

**Figure 4:**
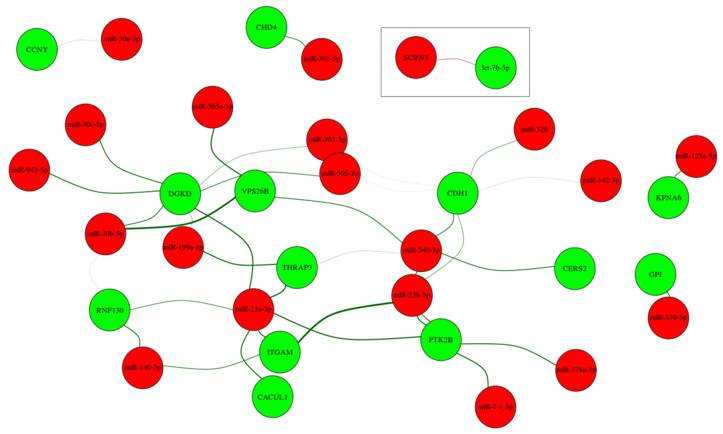
Network of the differentially expressed miRNA and their potential target mRNAs with inverse correlations and high differences in correlation between EA patients and healthy controls.

The miRNA-mRNA target analysis identified 14 target genes for the 19 miRNAs, which were used to construct the network shown in Figure 4. The width of the edge is determined by the correlation difference and the color shows the mode of interaction either higher-abundance level of mRNA and lower-abundance level of miRNA (Green) or the other way around (Red). Nodes are color-coded either for mRNA (Green) or miRNA (Red). The majority of the inverse correlated miRNA and transcripts were detected between the lower abundance level of miRNA and higher abundance level of mRNA, except for let-7b-5p, which showed a higher abundance level and their target gene, *SCRN3,* showed a lower abundance level. In the network, it is visible that some transcripts seem to be targeted by several miRNAs, whereas others just show interaction with one specific miRNA. Furthermore, some miRNAs seem to be regulating multiple transcript targets, e.g., miR-340-3p, miR-23b-3p, and miR-23a-3p.

### 3.7. Classification and Overrepresentation Enrichment Analysis

**Figure 5:**
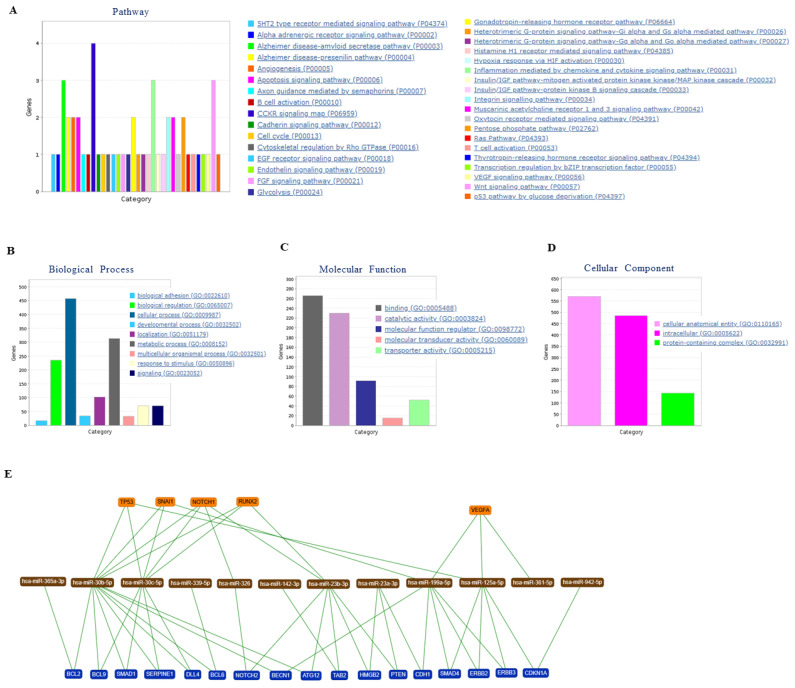
Pathways, Gene Ontology, and network enrichment analyses by using PANTHER and miRTargetLink tools for the identified protein-coding genes through mRNA microarray. Distribution of genes according to the (**A**) KEGG pathways, (**B**) biological process, (**C**) molecular function, (**D**) type of cellular component, and (**E**) network of the identified miRNAs, which have a binding site within the 3′UTR of the identified target mRNAs. MiRNAs are shown as brown nodes, genes targeted by at two miRNAs as blue, and genes targeted by at least three miRNAs as orange nodes.

To gain a more comprehensive insight into the regulation of miRNAs and mRNA, we performed network, comparative pathway, and gene ontology analyses. First, considering only the protein-coding genes, the differentially abundant mRNAs were enriched utilizing the PANTHER tool into GO terms, including biological process, molecular function, cellular component, as well as KEGG pathways. Pathway analysis identified 35 significant pathways (Figure 5A). Top pathways underlying the differentially abundant mRNAs are mostly involved in signal transduction and cellular interaction pathways. The biological processes of mRNAs were mainly enriched in biological adhesion regulation, localization, and cellular, developmental, and metabolic processes (Figure 5B). The enriched mRNAs in molecular function and cellular component were mainly associated with binding, catalytic activity, and transporter activity and cellular anatomical entity and intracellular, respectively (Figure 5C,D). Using miRTargetLink, we selected those miRNAs that showed lower abundance levels in patients with EA compared to HVs and also have a binding site within the 3′UTR region of the identified target genes, as shown in Figure 4. For these miRNAs, we constructed the target gene network, which is presented in Figure 5E. In this network, miRNAs are represented as brown nodes, genes targeted by two miRNAs in blue, and by three or more miRNAs in orange. Genes only targeted by a single miRNA were not represented. Our analysis highlighted five genes targeted by at least three miRNAs: *TP53*, *SNAI1*, *NOTCH1*, *RUNX2*, and *VEGFA.* These genes were targeted by seven miRNAs: hsa-miR-326, hsa-miR-23b-3p, hsa-miR-361-5p, hsa-miR-30c-5p, hsa-miR-30b-5p, hsa-miR-199a-5p, and hsa-miR-125a-5p. Additionally, 17 genes were targeted by at least two miRNAs are highlighted in blue (Figure 5E).

## 4. Discussion

In this study, we systematically analyzed, for the first time, the miRNA and mRNA abundance levels in blood samples collected from patients with EA and HVs. With miRNA and mRNA microarray profiling for 2549 miRNAs and 50,599 transcripts, respectively, we found 181 miRNAs and 64 transcripts whose abundance levels were significantly altered in the patients’ group compared to the HVs. Integrative analysis resulted in 37 interactions between the identified 19 miRNAs and 14 target mRNAs that showed fold change values ≥ 2.0 and ≥ 1.5 and significantly high correlation differences between the EA patient group and the HVs (Figure 4). These 19 miRNAs have binding sites within the 3′UTR region of the identified target genes. Abundance levels of certain miRNA and mRNA in the blood of EA patients differ from HVs (Figure 2A,B). These distinct differences in specific miRNA abundance patterns may lead to the discovery of novel biomarkers characteristic for EA patients. BNP expression and hematocrit (Hct) values have been previously identified to increase in patients with severe EA [29]. Thus, they are currently the only potential biomarkers because genetic factors related to EA are heterogeneous [30]. Standard diagnosis procedures are still relying on two-dimensional echocardiography, chest X-Ray, and electrocardiography [31] and could be facilitated with the use of specific biomarkers in the blood, such as miRNA or mRNA and/or both of them.

Pathway enrichment analysis of the deregulated mRNAs identified 35 significant pathways (Figure 5A). One of these pathways is the endothelin signaling pathway, in which Proline-rich tyrosine kinase 2 (*Pyk2*) plays an important role [32]. *Pyk2* is encoded by Protein Tyrosine Kinase 2 Beta (*PTK2B)* [33], which is part of our interaction network for EA. Deregulation of the endothelin axis, which consists of three peptides, two receptors, and one enzyme, can contribute to processes responsible for cardiovascular disease states including systemic and pulmonary hypertension and congestive heart failure [34]. Another significant pathway identified is the cholecystokinin receptor (*CCKR*) signaling pathway. This pathway is not only important in the digestive system [35] but one of its components, cholecystokinin (*CCK*), is an independent marker of cardiovascular mortality in plasma in elderly female patients [36]. The p53-pathway is enriched in our data as well, this pathway is one of the pathways activating apoptosis and autophagy, which in turn participate in the pathogenesis, development, and prognosis of coronary heart diseases [37]. Another pathway, the integrin signaling pathway, was observed among the significantly enriched pathways. This pathway mediates signals from the extracellular matrix to cells within the failing heart [38].

Using miRTargetLink a second target gene network was constructed (Figure 5E), in both network analyses the gene Cadherin 1 (*CDH1)* showed interactions with deregulated miRNAs and is, therefore, an interesting target for further analyses. *CDH1*, encoding for E-cadherin is involved in the maintenance of tissue architecture, as well as embryogenesis, and is associated with several congenital abnormalities [39]. It is not only involved in the Wnt-signaling pathway but also in the cadherin signaling pathway, which is enriched in our findings (Figure 5A). The Snail Family Transcriptional Repressor 1 (*SNAI1)* transcription factor, which is targeted by three deregulated miRNAs represses the transcription of E-cadherin by binding to the promoter [40], so this pathway seems to be strongly affected in EA patients. Other members of the cadherin, namely T-cadherin, have been previously associated with multiple conditions of cardio-metabolic disease and were suggested as diagnostic markers for early stages of cardiovascular disease [41]. Runt-related transcription factor 2 (*RUNX2*) (Figure 5E), which is a target for three altered miRNAs in patients with EA compared to HVs. It regulates cell proliferation and is one of the key transcription factors in the differentiation of mesenchymal stem cells [42] and its abundance level is regulated by the Notch signaling pathway [43].

Of the dysregulated miRNAs, many miRNAs have been reported to play a role in cardiovascular diseases and many others have not yet been reported to be related to any biological function in cardiovascular diseases and/or CHD-related manifestations. MiR-30 has been previously shown to play a regulatory role in the development of cardiac hypertrophy [44] and has already been proposed as a biomarker for heart failure [45]. Additionally, deregulated abundance level of miR-132 has been reported in humans with pathological cardiac remodeling [46]. In agreement with our finding, four miRNAs including miR-328, miR-210, miR-146a, and miR-142-5p, showed altered abundance levels in patients with chronic heart failure [47]. MicroRNA-199a-5p, which is deregulated in EA patients, mediates cardiomyocyte apoptosis, and is involved in the progression of heart failure [48]. Furthermore, miR-199a induces cardiac regeneration and improves cardiac function after myocardial infarction injury in mice [49]. Moreover, miR-199a-5p together with miR-125a-5p and miR-361-5p target the Vascular Endothelial Growth Factor A (*VEGFA)* (Figure 5E). *VEGFA* plays a functional role in mediating angiogenesis and is responsible for a variety of physio/pathological processes in cardiovascular diseases. A higher concentration of *VEGFA* was observed in patients with different classes of heart diseases and was correlated with disease severity, as well as unfavorable prognosis [50]. In our findings, the *VEGF* signaling pathway was one of the significantly enriched pathways suggesting that this pathway plays a biological function in the patients with EA with cardiovascular diseases and/or in CHD-related manifestations.

Previous studies suggested that cardiac functions are compromised if miRNAs let-7b-5p, miR-210-3p, and miR-331-3p are deregulated [51]. In agreement with our findings, these three miRNAs showed a significantly altered deregulated abundance level in EA compared to HVs. Specifically, let-7b-5p showed a higher abundance level in EA compared to HVs and has a binding site to Secernin 3 (*SCRN3)* mRNA target, which has not yet been reported to be related to any biological function in cardiovascular diseases. Other miRNAs showed a lower abundance level including miR-23a-3p, miR-19a-3p, miR-26a-5p, miR-29c-5p, and miR-125b-5p (Table 1). Of these miRNAs, miR-23a-3p has cardioprotective effects and mediates induction of angiogenesis in the ischemic heart after myocardial infarction, while the other three miRNAs enhance cardiomyocyte survival, function, and attenuate cardiac fibrosis [52]. In pathological response, a reduction of cardioprotective effects, due to the altered abundance level of these miRNAs, may possibly lead to EA.

Members of the miR-15 family have been reported to be deregulated and contribute to cardiomyocyte mitotic arrest during postnatal heart development, whereas in our study, miR-15b-3p is also deregulated. Lower abundance levels of miR-15b-3p, along with miR-128-3p, have been previously associated with cell cycle exit when upregulated during postnatal heart growth [49]. MicroRNA-145 was also reported to specifically target Frataxin (*FXN*), which is reported to regulate apoptosis and mitochondrial function, and was also reported to regulate the development of CHD [53]. The highly conversed cardiac and skeletal muscle-specific expression of miR-133 during development was observed to negatively regulate cardiomyocyte proliferation. Moreover, its altered abundance level in mutant mice led to ventricular-septal defects [54]. Similarly, miR-142 was observed to promote cardiomyocyte proliferation in embryonic, postnatal, and adult hearts [54]. Another two miRNAs, miR-93 and miR-148a, were reported to regulate target genes involved in cell survival, proliferation, metabolism, extracellular matrix turnover, and impaired proteosomal function [55], suggesting that these miRNAs play a distinct role during heart development and/or under pathological disorders [49]. Not only miRNAs but also the mRNA targets are involved in cardiovascular-related developmental processes. Specifically, the abundance level of Diacylglycerol Kinase Delta (*DGKD*) mRNA was inversely correlated with several differentially abundant miRNAs in our study (Figure 4). *DGKD* was suggested to be involved in the development and function of the central nervous system [56] and plays an important role in metabolic regulation and its altered abundance level is associated with epilepsy or Type II diabetes [57].

In this study, the analysis of miRNA and mRNA derived from blood samples, not from cardiac tissues, provides specific miRNA expression patterns that may lead to identifying novel biomarkers to characterize the anomaly, rather than providing information on underlying molecular mechanisms in EA patients. Since these patients with complex congenital cardiovascular anatomy are increasingly surviving surgical and therapeutic management at an early age, long-term cardiac morbidities and survival are more and more important in this regard. The analysis of patients’ heart tissues would be also a highly interesting objective. However, the analysis of heart tissue does not help to corroborate our findings on circulating miRNAs; as shown in several studies that the expression of miRNAs is highly tissue-specific [58,59]. Therefore, our findings on blood-based miRNAs will not readily relate to miRNA data generated for patients’ heart tissues. Additionally, our findings suggest communication between circulating RNAs and tissues-derived RNAs leading to the identified miRNA/mRNA signature in the blood of patients with EA. We assume that the detected miRNAs/mRNAs in EA patient samples in comparison to those from healthy volunteers have the potential as non-invasive biomarkers for facilitating the diagnosis. Nevertheless, it should be noted that the relationship between the blood-based circulating miRNAs/mRNAs and tissue-specific miRNAs/mRNAs remains still to be elucidated in EA patients. In other diseases, this relationship is already better understood as for example, in kidney-related diseases, due to the stable contact between urinary tract and kidney tissues [60]. In this study, most of the included patients were in asymptomatic cardiac conditions, and clinically manifested heart failure was not found in any of these patients. Certainly, the right heart shape and characteristic malformations of the tricuspid valve are associated with changes in the hemodynamic leading to right to left shunt under exercise in these patients. The left heart function in nearly all patients was normal. Although the assessment of the molecular mechanisms in the heart tissues, specifically, in the right heart, would be extremely interesting. only a few patients underwent heart surgery in the past. According to the significant differences in the miRNA and mRNA expression, we believe that the identified signature is related to the rare and characteristic malformations of the tricuspid valve and the associated other anatomical and functional abnormalities.

In conclusion, according to our results, we demonstrate for the first time a clear differentiation between patients with EA of the tricuspid valve and HVs using miRNA and mRNA expression profiling in blood. Since the identified miRNAs and mRNAs have also been reported in the context of several cardiac pathologies including congenital heart diseases it is legitimate to speculate about a possible role of the identified miRNAs for EA patients. Conclusive evidence of such a causal role of the identified miRNAs needs, however, to be established in independent experiments to identify, among others, the cells of origin for each of deregulated miRNA-mRNA pairs. Notably, the identified miRNAs and mRNAs may serve as a biomarker for EA. We would like to point out that this study also has a number of limitations. One important point is that the study population was small and heterogeneous since EA is a rare CHD and only 16 patients agreed to participate in this study. Thus, a multicenter study including a higher number of patients is necessary to identify the possible miRNA and/or mRNA marker for the severity of EA, familiar accumulation, the occurrence of non-compaction cardiomyopathy mainly involving the left ventricle, and other cardiac-related co-morbidities, like arrhythmia and right heart failure. An improved understanding of pathomechanisms underlying such rare diseases and the related alterations in interaction networks of miRNA and mRNA may to facilitate the better characterization of EA and other congenital heart and vessel malformations and may lead to progress in clinical management.

## Figures and Tables

**Table 1:** Significantly abundant miRNAs in the blood of patients with Ebstein’s anomaly compared to healthy controls as determined by microarray.

MicroRNA	Median Ebstein’s Anomaly	Median Controls	Fold Change	Regulation	Adjusted *p*-Value
hsa-miR-186-5p	4.47	6.92	0.17	Lower	3.63 × 10^−9^
hsa-miR-199a-5p	3.87	6.09	0.19	Lower	1.02 × 10^−7^
hsa-miR-142-3p	6.29	8.09	0.23	Lower	2.53 × 10^−5^
hsa-miR-148b-3p	2.85	4.59	0.24	Lower	1.02 × 10^−7^
hsa-miR-215-5p	6.38	8.19	0.24	Lower	0.00011
hsa-miR-340-3p	3.64	5.36	0.25	Lower	4.68 × 10^−6^
hsa-miR-128-3p	5.03	6.73	0.25	Lower	1.73 × 10^−5^
hsa-miR-15b-3p	1.66	3.56	0.25	Lower	1.58 × 10^−7^
hsa-miR-145-5p	2.77	4.75	0.27	Lower	1.76 × 10^−8^
hsa-miR-505-3p	2.55	4.16	0.27	Lower	1.19 × 10^−8^
hsa-miR-23b-3p	4.86	6.28	0.29	Lower	2.13 × 10^−5^
hsa-miR-942-5p	2.42	4.05	0.30	Lower	2.12 × 10^−7^
hsa-miR-365a-3p	3.31	4.93	0.30	Lower	1.19 × 10^−8^
hsa-miR-7-1-3p	1.80	3.56	0.31	Lower	1.19 × 10^−8^
hsa-miR-30b-5p	9.94	11.47	0.31	Lower	8.90 × 10^−6^
hsa-miR-23a-3p	7.73	9.17	0.31	Lower	3.88 × 10^−6^
hsa-miR-125b-5p	3.65	6.01	0.31	Lower	0.00201
hsa-miR-182-5p	2.69	4.48	0.32	Lower	1.62 × 10^−5^
hsa-miR-29c-5p	4.50	6.11	0.33	Lower	0.00023
hsa-miR-361-3p	5.36	6.78	0.34	Lower	2.74 × 10^−5^
hsa-miR-4732-3p	3.65	4.91	0.34	Lower	3.58 × 10^−6^
hsa-miR-454-5p	1.72	3.33	0.35	Lower	1.36 × 10^−7^
hsa-miR-30a-5p	3.03	4.83	0.35	Lower	0.00036
hsa-miR-378a-5p	3.86	5.04	0.36	Lower	3.30 × 10^−5^
hsa-miR-30e-3p	2.68	4.20	0.37	Lower	0.00019
hsa-miR-26a-5p	6.84	7.96	0.37	Lower	0.00244
hsa-miR-99b-5p	2.24	3.68	0.37	Lower	3.58 × 10^−6^
hsa-miR-5189-3p	1.90	3.10	0.38	Lower	0.00076
hsa-miR-326	2.90	4.48	0.40	Lower	2.37 × 10^−5^
hsa-miR-664a-3p	3.87	5.11	0.40	Lower	3.63 × 10^−9^
hsa-miR-93-3p	4.40	5.38	0.41	Lower	7.69 × 10^−5^
hsa-miR-30c-5p	9.58	10.67	0.41	Lower	3.09 × 10^−7^
hsa-miR-5690	1.74	2.93	0.42	Lower	3.58 × 10^−6^
hsa-miR-361-5p	4.88	5.79	0.42	Lower	0.00034
hsa-miR-3653-3p	4.15	4.96	0.43	Lower	0.00208
hsa-miR-140-3p	9.86	11.05	0.44	Lower	2.17 × 10^−7^
hsa-miR-339-5p	2.28	3.32	0.44	Lower	0.00014
hsa-miR-133b	1.62	2.62	0.44	Lower	0.00036
hsa-miR-148a-3p	3.29	4.22	0.44	Lower	0.00244
hsa-miR-146a-5p	2.71	3.79	0.44	Lower	2.49 × 10^−6^
hsa-miR-744-5p	2.12	3.28	0.45	Lower	1.65 × 10^−6^
hsa-miR-99a-5p	2.09	3.11	0.45	Lower	0.00025
hsa-miR-194-5p	6.94	7.54	0.47	Lower	0.00801
hsa-miR-1285-3p	1.81	2.92	0.47	Lower	3.61 × 10^−8^
hsa-miR-502-5p	2.37	3.50	0.48	Lower	0.00010
hsa-miR-550a-3p	6.68	7.80	0.48	Lower	0.00042
hsa-miR-191-5p	2.63	3.78	0.48	Lower	0.00011
hsa-miR-16-2-3p	3.65	4.59	0.50	Lower	0.00188
hsa-miR-125a-5p	4.16	5.22	0.50	Lower	0.00062
hsa-miR-5739	6.01	4.48	3.71	Higher	4.68 × 10^−6^
hsa-miR-638	4.77	3.31	3.04	Higher	3.05 × 10^−7^
hsa-miR-4459	6.84	5.55	2.92	Higher	5.09 × 10^−6^
hsa-miR-6089	8.00	6.68	2.90	Higher	4.63 × 10^−8^
hsa-miR-6165	4.76	3.51	2.86	Higher	2.76 × 10^−6^
hsa-miR-6749-5p	5.18	4.01	2.77	Higher	2.70 × 10^−5^
hsa-miR-6085	4.95	3.47	2.73	Higher	0.00025
hsa-miR-3162-5p	5.49	4.22	2.65	Higher	1.24 × 10^−6^
hsa-miR-7977	11.10	9.75	2.55	Higher	0.01260
hsa-miR-4728-5p	4.65	3.41	2.44	Higher	0.00075
hsa-miR-3656	4.77	3.43	2.42	Higher	0.00020
hsa-miR-4800-5p	3.91	2.14	2.39	Higher	0.00219
hsa-miR-6087	6.56	5.40	2.38	Higher	1.24 × 10^−6^
hsa-miR-7107-5p	3.59	1.99	2.34	Higher	0.00076
hsa-miR-6125	5.56	4.30	2.28	Higher	3.05 × 10^−7^
hsa-miR-7114-5p	4.17	3.10	2.25	Higher	0.00109
hsa-miR-4286	7.54	6.49	2.20	Higher	0.01379
hsa-miR-6869-5p	5.48	4.28	2.19	Higher	2.98 × 10^−6^
hsa-miR-4505	5.90	4.71	2.16	Higher	1.82 × 10^−5^
hsa-miR-210-3p	7.23	6.11	2.16	Higher	0.00056
hsa-miR-1202	4.63	3.60	2.14	Higher	2.76 × 10^−6^
hsa-let-7b-5p	11.47	10.25	2.12	Higher	4.72 × 10^−6^
hsa-miR-6740-5p	5.84	5.02	2.09	Higher	4.04 × 10^−6^
hsa-miR-2861	4.41	3.26	2.06	Higher	2.76 × 10^−6^
hsa-miR-7704	4.65	3.41	2.05	Higher	6.90 × 10^−6^
hsa-miR-1268b	4.20	3.31	2.05	Higher	0.00019
hsa-miR-4507	5.37	4.41	2.02	Higher	0.00224

An unpaired two-tailed t-test was used to calculate the *p*-value. Benjamini–Hochberg False Discovery Rate (FDR) correction was used to adjust the *p*-value. Significant changes in abundance levels are shown with an adjusted *p*-value < 0.05 and fold change (FC) ≥2.

**Table 2:** Significantly abundant transcripts in the blood of patients with Ebstein’s anomaly compared to healthy controls as determined by microarray.

Human Symbol	Gene ID	Median Ebstein’s Anomaly	Median Controls	Fold Change	Regulation	Adjusted *p*-Value
KANK4 (KN motif and ankyrin repeat domains 4)	163782	7.59	6.45	2.71	Higher	0.0400
ADGRE4P (adhesion G protein-coupled receptor E4, pseudogene)	326342	5.10	3.58	2.53	Higher	0.0383
KCNG1 (potassium voltage-gated channel modifier subfamily G member 1)	3755	6.41	5.22	2.48	Higher	0.0383
IGF2R (insulin-like growth factor 2 receptor)	3482	10.29	9.49	2.06	Higher	0.0404
BAZ2A (bromodomain adjacent to zinc finger domain 2A)	11176	8.68	7.70	1.95	Higher	0.0082
BACE2 (beta-secretase 2)	25825	8.92	8.11	1.91	Higher	0.0400
PGD (phosphogluconate dehydrogenase)	5226	11.50	10.55	1.83	Higher	0.0404
KDM1A (lysine demethylase 1A)	23028	5.86	5.12	1.82	Higher	0.0065
RIOK1 (RIO kinase 1)	83732	7.42	6.75	1.81	Higher	0.0161
PRPF38B (pre-mRNA processing factor 38B)	55119	7.36	6.60	1.73	Higher	0.0383
ITGAM (integrin subunit alpha M)	3684	10.80	9.92	1.72	Higher	0.0383
CCNY (cyclin Y)	219771	9.65	8.84	1.68	Higher	0.0383
ZFP91 (ZFP91 zinc finger protein, atypical E3 ubiquitin ligase)	80829	8.40	7.64	1.68	Higher	0.0169
ARID1A (AT-rich interaction domain 1A)	8289	8.19	7.52	1.66	Higher	0.0457
LASP1 (LIM and SH3 protein 1)	3927	11.34	10.78	1.59	Higher	0.0065
VPS35 (VPS35 retromer complex component)	55737	9.87	9.24	1.59	Higher	0.0082
PAK1 (p21 (RAC1) activated kinase 1)	5058	11.68	11.15	1.55	Higher	0.0383
RBM23 (RNA binding motif protein 23)	55147	8.40	7.81	1.54	Higher	0.0400
CUX1 (cut like homeobox 1)	1523	8.89	8.29	1.53	Higher	0.0457
WDR1 (WD repeat domain 1)	9948	9.08	8.58	1.52	Higher	0.0090
CACUL1 (CDK2 associated cullin domain 1)	143384	8.47	7.87	1.52	Higher	0.0224
CHD4 (chromodomain helicase DNA binding protein 4)	1108	9.15	8.68	1.51	Higher	0.0400
PSME3 (proteasome activator subunit 3)	10197	9.16	8.57	1.51	Higher	0.0383
VPS26B (VPS26, retromer complex component B)	112936	9.30	8.78	1.51	Higher	0.0191
IL17RA (interleukin 17 receptor A)	23765	11.95	11.50	1.50	Higher	0.0383
SCRN3 (secernin 3)	79634	5.19	6.43	0.44	Lower	0.0400
SLC9A4 (solute carrier family 9 member A4)	389015	8.48	9.15	0.59	Lower	0.0383
PRKAA2 (protein kinase AMP-activated catalytic subunit alpha 2)	5563	4.04	4.67	0.59	Lower	0.0404
LCN8 (lipocalin 8)	138307	4.35	4.98	0.60	Lower	0.0404

An un-paired two-tailed *t*-test was used to calculate the *p*-value. Benjamini–Hochberg False Discovery Rate (FDR) correction was used to adjust the *p*-value. Significant changes in abundance levels are shown with an adjusted *p*-value < 0.05 and fold change (FC) ≥ 2.

**Table 3:** Significantly dysregulated miRNA in the blood of patients with familial Ebstein’s anomaly compared to non-familial Ebstein’s anomaly patients.

Micro-RNA	Median Familial (*n* = 4) Ebstein’s Anomaly	Median Non-Familial (*n* = 12) Ebstein’s Anomaly	Fold Change	Regulation	Adjusted *p*-Value
hsa-miR-1202	4.17	4.90	0.59	Lower	0.0186
hsa-miR-3162-5p	5.04	5.63	0.60	Lower	0.0186
hsa-miR-326	4.01	2.73	2.15	Higher	0.0186
hsa-miR-550a-3p	7.52	6.31	2.26	Higher	0.0186
hsa-miR-629-3p	3.96	2.75	2.08	Higher	0.0186
hsa-miR-6085	3.90	5.23	0.39	Lower	0.0217
hsa-miR-6165	4.42	5.21	0.53	Lower	0.0217
hsa-miR-378a-5p	4.54	3.51	2.29	Higher	0.0390

An unpaired two-tailed *t*-test was used to calculate the *p*-value. Benjamini–Hochberg False Discovery Rate (FDR) correction was used to adjust the *p*-value. Significant changes in abundance levels are shown with an adjusted *p*-value < 0.05.

## Data Availability

All relevant data are within the paper and its Supporting Information files.

## Supplementary Materials

The following are available online at https://www.mdpi.com/article/10.3390/cells10051066/s1. Table S1: Clinical Characteristics of included Ebstein’s anomaly patients. Table S2: Significantly abundant miRNAs in the blood of patients with Ebstein’s anomaly compared to healthy controls as determined by microarray. Table S3: Significantly abundant transcripts in the blood of patients with Ebstein’s anomaly compared to healthy controls as determined by microarray. Table S4: Significant inverse correlation between the identified miRNA and their potential target mRNAs.
