# Supplementary material for: Integrated microRNA and mRNA Expression Profiling Identifies Novel Targets and Networks Associated with Ebstein’s Anomaly
Source: Cells. 2021 Apr 30;10(5):1066. doi: 10.3390/cells10051066 (PMC8146150; doi:10.3390/cells10051066)
Supplement: Supplementary file 1 [file cells-10-01066-s001.zip › cells-1176291-supplementary.pdf]

SUPPLEMENTARY TABLE 1: Clinical Characteristics of included Ebstein's anomaly patients

Parameters	Ebstein's Anomaly (n = 16)
Age (Years)	25.8 ± 12.4
Weight (kg)	63.7 ± 14.00
Lenght (cm)	165 ± 8.12
Body Mass Index (BMI, kg/m²)	1.63 ± 0.28
Systolic Blood Pressure (SBP, mmHg)	120 ± 9.69
Diastolic Blood Pressure (DBP, mmHg)	67.4 ± 9.32
Heart Rate (bpm)	71.9 ± 10.80
**Severity of Ebstein's anomaly based on Carpentier criteria**	
• Mild	5/16 (31.25 %)
• Moderate	9/16 (56.25 %)
• Sever	2/16 12.50 %)
**New York Heart Association (NYHA)**	
• NYHA Class I	8/16 (50.0 %)
• NYHA Class II	3/16 (18.75 %)
• NYHA Class III	5/16 (31.25 %)
**Other parameters**	
Familial Ebstein's anomaly	4/16 (25.0 %)
Compaction cardiomyopathy of the left ventricle	8/16 (50.0 %)
Supraventricular arrhythmias	7/16 (43.75 %)
Atrial septal defect (ASD)	7/16 (43.75 %)
Reconstructive surgery for the tricuspid valve	1/16 (6.25 %)

- Mean ± standard deviation was used

SUPPLEMENTARY TABLE 2: Significantly abundant miRNAs in the blood of patients with Ebstein's anomaly compared to healthy controls as determined by microarray.

MicroRNA	Median Ebstein's anomaly	Median Controls	Fold change	Regulation	*P*-value	Adjusted *P*-value	AUC
hsa-miR-186-5p	4.47	6.92	0.17	Lower	2.00E-11	3.63E-09	0.01
hsa-miR-199a-5p	3.87	6.09	0.19	Lower	4.23E-09	1.02E-07	0.01
hsa-miR-142-3p	6.29	8.09	0.23	Lower	5.14E-06	2.53E-05	0.05
hsa-miR-148b-3p	2.85	4.59	0.24	Lower	4.28E-09	1.02E-07	0.01
hsa-miR-215-5p	6.38	8.19	0.24	Lower	2.66E-05	0.00011	0.03
hsa-miR-340-3p	3.64	5.36	0.25	Lower	6.15E-07	4.68E-06	0.02
hsa-miR-128-3p	5.03	6.73	0.25	Lower	3.19E-06	1.73E-05	0.01

hsa-miR-15b-3p	1.66	3.56	0.25	Lower	7.87E-09	1.58E-07	0.01
hsa-miR-145-5p	2.77	4.75	0.27	Lower	4.04E-10	1.76E-08	0.01
hsa-miR-505-3p	2.55	4.16	0.27	Lower	2.16E-10	1.19E-08	0.01
hsa-miR-23b-3p	4.86	6.28	0.29	Lower	4.16E-06	2.13E-05	0.01
hsa-miR-942-5p	2.42	4.05	0.30	Lower	1.14E-08	2.12E-07	0.01
hsa-miR-365a-3p	3.31	4.93	0.30	Lower	2.28E-10	1.19E-08	0.01
hsa-miR-7-1-3p	1.80	3.56	0.31	Lower	1.65E-10	1.19E-08	0.01
hsa-miR-30b-5p	9.94	11.47	0.31	Lower	1.47E-06	8.90E-06	0.01
hsa-miR-23a-3p	7.73	9.17	0.31	Lower	4.76E-07	3.88E-06	0.01
hsa-miR-125b-5p	3.65	6.01	0.31	Lower	0.00082	0.00201	0.17
hsa-miR-182-5p	2.69	4.48	0.32	Lower	2.92E-06	1.62E-05	0.04
hsa-miR-29c-5p	4.50	6.11	0.33	Lower	6.20E-05	0.00023	0.10
hsa-miR-361-3p	5.36	6.78	0.34	Lower	5.77E-06	2.74E-05	0.02
hsa-miR-4732-3p	3.65	4.91	0.34	Lower	4.25E-07	3.58E-06	0.01
hsa-miR-454-5p	1.72	3.33	0.35	Lower	6.24E-09	1.36E-07	0.00
hsa-miR-30a-5p	3.03	4.83	0.35	Lower	0.00011	0.00036	0.11
hsa-miR-378a-5p	3.86	5.04	0.36	Lower	7.09E-06	3.30E-05	0.03
hsa-miR-30e-3p	2.68	4.20	0.37	Lower	4.77E-05	0.00019	0.09
hsa-miR-26a-5p	6.84	7.96	0.37	Lower	0.00106	0.00244	0.13
hsa-miR-99b-5p	2.24	3.68	0.37	Lower	4.20E-07	3.58E-06	0.03
hsa-miR-5189-3p	1.90	3.10	0.38	Lower	0.00028	0.00076	0.13
hsa-miR-326	2.90	4.48	0.40	Lower	4.72E-06	2.37E-05	0.05
hsa-miR-664a-3p	3.87	5.11	0.40	Lower	2.78E-11	3.63E-09	0.00
hsa-miR-93-3p	4.40	5.38	0.41	Lower	1.71E-05	7.69E-05	0.01
hsa-miR-30c-5p	9.58	10.67	0.41	Lower	2.13E-08	3.09E-07	0.01
hsa-miR-5690	1.74	2.93	0.42	Lower	4.08E-07	3.58E-06	0.01
hsa-miR-361-5p	4.88	5.79	0.42	Lower	9.59E-05	0.00034	0.01
hsa-miR-3653-3p	4.15	4.96	0.43	Lower	0.00085	0.00208	0.16
hsa-miR-140-3p	9.86	11.05	0.44	Lower	1.25E-08	2.17E-07	0.01
hsa-miR-339-5p	2.28	3.32	0.44	Lower	3.59E-05	0.00014	0.08
hsa-miR-133b	1.62	2.62	0.44	Lower	0.00011	0.00036	0.08
hsa-miR-148a-3p	3.29	4.22	0.44	Lower	0.00107	0.00244	0.12
hsa-miR-146a-5p	2.71	3.79	0.44	Lower	2.29E-07	2.49E-06	0.01
hsa-miR-744-5p	2.12	3.28	0.45	Lower	1.45E-07	1.65E-06	0.06
hsa-miR-99a-5p	2.09	3.11	0.45	Lower	6.74E-05	0.00025	0.07
hsa-miR-194-5p	6.94	7.54	0.47	Lower	0.00463	0.00801	0.08
hsa-miR-1285-3p	1.81	2.92	0.47	Lower	1.11E-09	3.61E-08	0.01
hsa-miR-502-5p	2.37	3.50	0.48	Lower	2.33E-05	0.00010	0.09
hsa-miR-550a-3p	6.68	7.80	0.48	Lower	0.00014	0.00042	0.12
hsa-miR-191-5p	2.63	3.78	0.48	Lower	2.65E-05	0.00011	0.07
hsa-miR-16-2-3p	3.65	4.59	0.50	Lower	0.00076	0.00188	0.14
hsa-miR-125a-5p	4.16	5.22	0.50	Lower	0.00022	0.00062	0.11

hsa-miR-19a-3p	5.68	6.49	0.50	Lower	0.03067	0.04472	0.28
hsa-miR-30e-5p	5.31	6.33	0.50	Lower	0.00034	0.00091	0.12
hsa-miR-222-3p	3.06	3.85	0.51	Lower	8.37E-05	0.00030	0.09
hsa-miR-30d-5p	8.61	9.58	0.51	Lower	0.00022	0.00061	0.13
hsa-miR-27a-3p	3.35	4.15	0.51	Lower	0.00256	0.00507	0.18
hsa-miR-423-3p	2.40	3.11	0.51	Lower	9.36E-07	6.11E-06	0.02
hsa-miR-501-3p	3.35	4.25	0.52	Lower	2.75E-05	0.00011	0.06
hsa-miR-324-5p	5.45	6.49	0.52	Lower	0.00044	0.00112	0.13
hsa-miR-3200-3p	1.72	2.75	0.53	Lower	8.97E-07	6.11E-06	0.04
hsa-miR-532-3p	7.76	8.36	0.53	Lower	0.00054	0.00139	0.11
hsa-miR-192-5p	8.76	9.17	0.54	Lower	0.02262	0.03452	0.19
hsa-miR-1271-5p	1.75	2.56	0.54	Lower	1.41E-05	6.45E-05	0.07
hsa-miR-296-5p	2.46	3.21	0.54	Lower	1.50E-06	8.90E-06	0.01
hsa-miR-151a-3p	6.32	7.14	0.54	Lower	0.00099	0.00232	0.14
hsa-miR-28-5p	3.22	4.01	0.54	Lower	0.00036	0.00095	0.13
hsa-miR-223-3p	12.78	13.69	0.56	Lower	0.00175	0.00378	0.15
hsa-miR-500a-3p	4.96	5.74	0.57	Lower	0.00411	0.00730	0.14
hsa-miR-142-5p	3.98	4.57	0.57	Lower	0.00113	0.00257	0.16
hsa-miR-501-5p	3.95	4.66	0.58	Lower	0.00065	0.00163	0.10
hsa-miR-4659a-3p	1.71	2.60	0.59	Lower	0.00015	0.00046	0.11
hsa-miR-362-5p	5.36	6.21	0.60	Lower	0.00214	0.00439	0.14
hsa-miR-502-3p	5.28	5.98	0.60	Lower	0.01365	0.02133	0.18
hsa-miR-500a-5p	3.37	4.16	0.60	Lower	0.00407	0.00727	0.15
hsa-miR-331-3p	10.67	11.05	0.61	Lower	0.01733	0.02677	0.20
hsa-miR-484	8.52	9.17	0.61	Lower	9.97E-05	0.00034	0.08
hsa-miR-629-5p	3.01	3.64	0.62	Lower	0.00195	0.00407	0.15
hsa-miR-625-5p	5.19	5.61	0.62	Lower	0.01016	0.01628	0.15
hsa-miR-4323	3.32	3.86	0.64	Lower	0.00238	0.00482	0.20
hsa-miR-151a-5p	8.99	9.36	0.65	Lower	0.00559	0.00935	0.21
hsa-miR-29b-3p	3.37	3.90	0.65	Lower	0.02921	0.04283	0.22
hsa-let-7d-3p	1.56	2.12	0.67	Lower	0.00014	0.00043	0.03
hsa-miR-454-3p	2.22	2.79	0.68	Lower	0.03089	0.04479	0.23
hsa-miR-29a-3p	6.22	6.86	0.68	Lower	0.00682	0.01126	0.18
hsa-miR-664b-3p	4.62	5.25	0.68	Lower	0.00037	0.00097	0.12
hsa-miR-328-3p	2.96	3.39	0.70	Lower	0.00368	0.00670	0.17
hsa-miR-6803-3p	2.02	2.42	0.70	Lower	0.00125	0.00274	0.11
hsa-miR-151b	8.24	8.65	0.72	Lower	0.02763	0.04121	0.24
hsa-miR-132-3p	3.47	3.82	0.72	Lower	0.02757	0.04121	0.26
hsa-miR-342-3p	8.80	9.27	0.72	Lower	0.00121	0.00269	0.16
hsa-miR-6511b-3p	1.84	2.22	0.74	Lower	0.00316	0.00606	0.10
hsa-miR-425-5p	11.47	11.62	0.82	Lower	0.00369	0.00670	0.19
hsa-miR-5739	6.01	4.48	3.71	Higher	6.28E-07	4.68E-06	0.99

hsa-miR-638	4.77	3.31	3.04	Higher	1.99E-08	3.05E-07	1.00
hsa-miR-4459	6.84	5.55	2.92	Higher	7.22E-07	5.09E-06	0.96
hsa-miR-6089	8.00	6.68	2.90	Higher	1.60E-09	4.63E-08	1.00
hsa-miR-6165	4.76	3.51	2.86	Higher	2.86E-07	2.76E-06	0.97
hsa-miR-6749-5p	5.18	4.01	2.77	Higher	5.58E-06	2.70E-05	0.98
hsa-miR-6085	4.95	3.47	2.73	Higher	6.58E-05	0.00025	0.90
hsa-miR-3162-5p	5.49	4.22	2.65	Higher	9.67E-08	1.24E-06	0.99
hsa-miR-7977	11.10	9.75	2.55	Higher	0.00769	0.01260	0.82
hsa-miR-4728-5p	4.65	3.41	2.44	Higher	0.00027	0.00075	0.90
hsa-miR-3656	4.77	3.43	2.42	Higher	5.31E-05	0.00020	0.87
hsa-miR-4800-5p	3.91	2.14	2.39	Higher	0.00091	0.00219	0.85
hsa-miR-6087	6.56	5.40	2.38	Higher	9.96E-08	1.24E-06	1.00
hsa-miR-7107-5p	3.59	1.99	2.34	Higher	0.00028	0.00076	0.87
hsa-miR-6125	5.56	4.30	2.28	Higher	1.97E-08	3.05E-07	0.99
hsa-miR-7114-5p	4.17	3.10	2.25	Higher	0.00042	0.00109	0.88
hsa-miR-4286	7.54	6.49	2.20	Higher	0.00856	0.01379	0.80
hsa-miR-6869-5p	5.48	4.28	2.19	Higher	3.20E-07	2.98E-06	0.97
hsa-miR-4505	5.90	4.71	2.16	Higher	3.48E-06	1.82E-05	0.93
hsa-miR-210-3p	7.23	6.11	2.16	Higher	0.00019	0.00056	0.90
hsa-miR-1202	4.63	3.60	2.14	Higher	2.71E-07	2.76E-06	0.98
hsa-let-7b-5p	11.47	10.25	2.12	Higher	6.51E-07	4.72E-06	0.95
hsa-miR-6740-5p	5.84	5.02	2.09	Higher	5.10E-07	4.04E-06	1.00
hsa-miR-2861	4.41	3.26	2.06	Higher	2.82E-07	2.76E-06	0.99
hsa-miR-7704	4.65	3.41	2.05	Higher	1.08E-06	6.90E-06	0.94
hsa-miR-1268b	4.20	3.31	2.05	Higher	4.92E-05	0.00019	0.92
hsa-miR-4507	5.37	4.41	2.02	Higher	0.00093	0.00224	0.86
hsa-miR-939-5p	3.95	2.92	1.96	Higher	1.78E-05	7.87E-05	0.92
hsa-let-7i-5p	9.42	8.36	1.95	Higher	0.00242	0.00486	0.82
hsa-miR-6124	4.75	4.01	1.95	Higher	2.06E-06	1.17E-05	1.00
hsa-miR-6879-5p	6.11	5.31	1.95	Higher	3.36E-05	0.00014	0.95
hsa-miR-6803-5p	6.16	4.96	1.95	Higher	0.00120	0.00269	0.83
hsa-miR-4713-3p	5.45	4.80	1.91	Higher	0.00013	0.00041	0.92
hsa-miR-4530	5.07	4.11	1.88	Higher	3.45E-06	1.82E-05	0.96
hsa-miR-4721	3.28	2.58	1.88	Higher	9.72E-05	0.00034	0.90
hsa-miR-8069	10.98	10.02	1.87	Higher	0.00487	0.00836	0.80
hsa-miR-6891-5p	3.01	2.03	1.82	Higher	5.26E-08	7.23E-07	0.95
hsa-miR-7110-5p	3.04	2.06	1.81	Higher	0.01273	0.02002	0.80
hsa-miR-3665	4.10	3.21	1.80	Higher	1.80E-06	1.04E-05	0.94
hsa-miR-5001-5p	2.68	1.93	1.79	Higher	9.35E-07	6.11E-06	0.94
hsa-miR-6875-5p	7.63	7.08	1.79	Higher	0.00256	0.00507	0.81
hsa-miR-4669	2.90	1.89	1.75	Higher	0.00022	0.00061	0.88
hsa-miR-1268a	3.55	2.60	1.74	Higher	0.00370	0.00670	0.86

hsa-miR-6780b-5p	5.75	5.25	1.72	Higher	0.00580	0.00964	0.80
hsa-miR-16-5p	11.85	10.91	1.71	Higher	0.02001	0.03072	0.76
hsa-miR-3198	4.80	4.18	1.70	Higher	0.00183	0.00387	0.88
hsa-miR-4716-3p	4.13	3.44	1.70	Higher	0.00123	0.00273	0.91
hsa-miR-4787-5p	2.92	2.17	1.70	Higher	1.27E-06	7.91E-06	0.93
hsa-miR-7975	14.37	14.37	1.70	Higher	0.01491	0.02316	0.73
hsa-miR-6807-5p	3.23	2.60	1.68	Higher	0.00011	0.00036	0.89
hsa-miR-5194	3.71	3.20	1.68	Higher	0.00360	0.00667	0.87
hsa-miR-6734-5p	4.38	3.76	1.67	Higher	0.00226	0.00461	0.89
hsa-miR-564	3.15	2.26	1.67	Higher	0.00318	0.00606	0.83
hsa-miR-6717-5p	5.20	4.73	1.66	Higher	0.00438	0.00767	0.87
hsa-miR-6131	5.06	4.46	1.66	Higher	0.00285	0.00554	0.85
hsa-miR-320b	9.53	9.12	1.66	Higher	0.00100	0.00232	0.83
hsa-miR-197-5p	6.07	5.28	1.65	Higher	8.41E-05	0.00030	0.92
hsa-miR-6769b-5p	2.94	1.92	1.64	Higher	0.00326	0.00616	0.78
hsa-miR-1305	4.52	3.98	1.64	Higher	0.00551	0.00929	0.87
hsa-miR-6724-5p	3.01	2.15	1.63	Higher	0.00011	0.00036	0.88
hsa-miR-3195	3.93	3.25	1.63	Higher	0.02559	0.03861	0.80
hsa-miR-4687-3p	3.87	3.19	1.63	Higher	6.95E-10	2.59E-08	0.99
hsa-miR-1587	3.30	2.62	1.63	Higher	0.00182	0.00387	0.80
hsa-miR-4299	6.78	5.83	1.62	Higher	0.02558	0.03861	0.75
hsa-miR-5581-5p	4.12	3.48	1.62	Higher	0.00537	0.00910	0.89
hsa-miR-6127	7.87	7.27	1.60	Higher	0.00350	0.00653	0.80
hsa-miR-6767-5p	3.95	3.33	1.60	Higher	0.00184	0.00388	0.89
hsa-miR-4442	4.82	4.20	1.57	Higher	0.00016	0.00047	0.91
hsa-miR-4281	6.03	5.17	1.57	Higher	0.00097	0.00230	0.87
hsa-miR-3679-5p	3.43	2.72	1.57	Higher	0.00020	0.00058	0.88
hsa-miR-5088-5p	3.42	3.01	1.56	Higher	0.00338	0.00634	0.86
hsa-miR-1915-3p	3.41	2.76	1.53	Higher	0.00013	0.00041	0.89
hsa-miR-320d	9.70	9.36	1.51	Higher	0.00429	0.00756	0.77
hsa-miR-7847-3p	3.73	3.08	1.50	Higher	0.00157	0.00341	0.85
hsa-miR-6893-5p	2.74	2.09	1.50	Higher	0.00380	0.00683	0.83
hsa-miR-1914-3p	4.37	3.83	1.49	Higher	0.00528	0.00901	0.85
hsa-miR-1207-5p	4.32	3.79	1.47	Higher	0.00058	0.00148	0.86
hsa-miR-7152-3p	4.00	3.61	1.47	Higher	0.02842	0.04190	0.79
hsa-miR-320a	8.71	8.29	1.46	Higher	0.00299	0.00578	0.81
hsa-miR-4497	3.12	2.41	1.42	Higher	0.00854	0.01379	0.80
hsa-miR-4788	6.84	6.11	1.41	Higher	0.01100	0.01740	0.77
hsa-miR-6088	4.31	3.83	1.41	Higher	1.33E-07	1.57E-06	0.96
hsa-miR-320c	9.05	8.61	1.41	Higher	0.00259	0.00509	0.81
hsa-miR-4653-3p	3.81	3.41	1.38	Higher	0.02830	0.04190	0.80
hsa-miR-4672	3.16	2.48	1.37	Higher	0.00773	0.01260	0.81

hsa-miR-4466	3.77	3.25	1.37	Higher	9.78E-05	0.00034	0.89
hsa-miR-6090	5.88	5.31	1.36	Higher	0.00210	0.00435	0.79
hsa-miR-320e	9.23	8.99	1.36	Higher	0.01077	0.01714	0.77
hsa-miR-1225-5p	4.54	4.13	1.35	Higher	0.00017	0.00050	0.86
hsa-miR-3960	6.82	6.53	1.29	Higher	0.03130	0.04513	0.70
hsa-miR-6800-5p	3.78	3.41	1.24	Higher	0.00458	0.00797	0.86

- Un-paired two-tailed t test was used to calculate the *P*-value.
- Benjamini-Hochberg False Discovery Rate (FDR) correction was used to adjust the *P*-value.
- Significant changes in abundance levels are shown with an adjusted *P*-value <0.05
- AUC, Area under the curve

Supplementary Table 3: Significantly abundant transcripts in the blood of patients with Ebstein's anomaly compared to healthy controls as determined by microarray.

Human Symbol	Gene ID	Median Ebstein's anomaly	Median Controls	Fold change	Regulation	*P*-value	Adjusted *P*-value	AUC
KANK4 (KN motif and ankyrin repeat domains 4)	163782	7.59	6.45	2.71	Higher	6.07E-05	0.0400	0.92
ADGRE4P (adhesion G protein-coupled receptor E4, pseudogene)	326342	5.10	3.58	2.53	Higher	4.81E-05	0.0383	0.89
KCNG1 (potassium voltage-gated channel modifier subfamily G member 1)	3755	6.41	5.22	2.48	Higher	4.07E-05	0.0383	0.88
IGF2R (insulin like growth factor 2 receptor)	3482	10.29	9.49	2.06	Higher	6.59E-05	0.0404	0.91
BAZ2A (bromodomain adjacent to zinc finger domain 2A)	11176	8.68	7.70	1.95	Higher	1.45E-06	0.0082	0.95
BACE2 (beta-secretase 2)	25825	8.92	8.11	1.91	Higher	5.80E-05	0.0400	0.90
PGD (phosphogluconate dehydrogenase)	5226	11.50	10.55	1.83	Higher	6.82E-05	0.0404	0.88
KDM1A (lysine demethylase 1A)	23028	5.86	5.12	1.82	Higher	4.82E-07	0.0065	0.97
RIOK1 (RIO kinase 1)	83732	7.42	6.75	1.81	Higher	5.43E-06	0.0161	0.97
PRPF38B (pre-mRNA processing factor 38B)	55119	7.36	6.60	1.73	Higher	3.38E-05	0.0383	0.89
ITGAM (integrin subunit alpha M)	3684	10.80	9.92	1.72	Higher	4.65E-05	0.0383	0.88
CCNY (cyclin Y)	219771	9.65	8.84	1.68	Higher	3.68E-05	0.0383	0.90
ZFP91 (ZFP91 zinc finger protein, atypical E3 ubiquitin ligase)	80829	8.40	7.64	1.68	Higher	7.49E-06	0.0169	0.92
ARID1A (AT-rich interaction domain 1A)	8289	8.19	7.52	1.66	Higher	0.0001	0.0457	0.90
LASP1 (LIM and SH3 protein 1)	3927	11.34	10.78	1.59	Higher	3.42E-07	0.0065	0.95
VPS35 (VPS35 retromer complex component)	55737	9.87	9.24	1.59	Higher	1.52E-06	0.0082	0.96
PAK1 (p21 (RAC1) activated kinase 1)	5058	11.68	11.15	1.55	Higher	4.00E-05	0.0383	0.92
RBM23 (RNA binding motif protein 23)	55147	8.40	7.81	1.54	Higher	6.04E-05	0.0400	0.90
CUX1 (cut like homeobox 1)	1523	8.89	8.29	1.53	Higher	0.0001	0.0457	0.87
WDR1 (WD repeat domain 1)	9948	9.08	8.58	1.52	Higher	2.34E-06	0.0090	0.95

CACUL1 (CDK2 associated cullin domain 1)	143384	8.47	7.87	1.52	Higher	1.24E-05	0.0224	0.90
CHD4 (chromodomain helicase DNA binding protein 4)	1108	9.15	8.68	1.51	Higher	5.41E-05	0.0400	0.90
PSME3 (proteasome activator subunit 3)	10197	9.16	8.57	1.51	Higher	3.09E-05	0.0383	0.92
VPS26B (VPS26, retromer complex component B)	112936	9.30	8.78	1.51	Higher	9.21E-06	0.0191	0.95
IL17RA (interleukin 17 receptor A)	23765	11.95	11.50	1.50	Higher	3.84E-05	0.0383	0.90
RNF130 (ring finger protein 130)	55819	11.45	10.83	1.49	Higher	2.24E-06	0.0090	0.94
ATG9B (autophagy related 9B)	285973	3.87	3.34	1.47	Higher	6.23E-06	0.0161	0.93
PSAP (prosaposin)	5660	12.44	11.89	1.45	Higher	8.62E-05	0.0418	0.87
PTK2B (protein tyrosine kinase 2 beta)	2185	12.47	11.81	1.44	Higher	0.0001	0.0472	0.90
FOXN3 (forkhead box N3)	1112	9.57	8.99	1.44	Higher	7.26E-05	0.0404	0.90
CDH1 (cadherin 1)	999	3.67	3.07	1.44	Higher	8.07E-05	0.0411	0.86
GPI (glucose-6-phosphate isomerase)	2821	12.41	11.96	1.44	Higher	3.70E-05	0.0383	0.89
THRAP3 (thyroid hormone receptor associated protein 3)	9967	11.08	10.56	1.43	Higher	4.39E-05	0.0383	0.88
GNL2 (G protein nucleolar 2)	29889	8.41	7.97	1.43	Higher	7.18E-05	0.0404	0.90
SH3KBP1 (SH3 domain containing kinase binding protein 1)	30011	11.10	10.44	1.42	Higher	5.30E-06	0.0161	0.91
PWP1 (PWP1 homolog, endonuclein)	11137	10.60	10.01	1.41	Higher	8.67E-05	0.0418	0.88
PRKCD (protein kinase C delta)	5580	11.20	10.76	1.40	Higher	5.86E-05	0.0400	0.88
ZNF24 (zinc finger protein 24)	7572	9.78	9.20	1.40	Higher	6.45E-05	0.0404	0.88
CERS2 (ceramide synthase 2)	29956	9.99	9.48	1.38	Higher	1.24E-05	0.0224	0.92
DGKD (diacylglycerol kinase delta)	8527	10.94	10.48	1.36	Higher	0.0001	0.0464	0.87
MKLN1-AS (MKLN1 antisense RNA)	100506881	3.73	3.32	1.34	Higher	7.85E-05	0.0408	0.88
KPNA6 (karyopherin subunit alpha 6)	23633	10.21	9.74	1.33	Higher	5.62E-05	0.0400	0.89
DHX16 (DEAH-box helicase 16)	8449	9.52	9.01	1.30	Higher	3.10E-05	0.0383	0.90
HDAC6 (histone deacetylase 6)	10013	8.72	8.45	1.26	Higher	4.22E-05	0.0383	0.90
GEMIN7 (gem nuclear organelle associated protein 7)	79760	8.79	9.26	0.73	Lower	3.94E-05	0.0383	0.10
ABCC6 (ATP binding cassette subfamily C member 6)	368	4.99	5.56	0.70	Lower	4.26E-05	0.0383	0.12
CREB3L4 (cAMP responsive element binding protein 3 like 4)	148327	7.55	8.17	0.68	Lower	7.20E-05	0.0404	0.13
LCN8 (lipocalin 8)	138307	4.35	4.98	0.60	Lower	7.54E-05	0.0404	0.07
PRKAA2 (protein kinase AMP-activated catalytic subunit alpha 2)	5563	4.04	4.67	0.59	Lower	7.44E-05	0.0404	0.04
SLC9A4 (solute carrier family 9 member A4)	389015	8.48	9.15	0.59	Lower	2.44E-05	0.0383	0.03
SCRN3 (secernin 3)	79634	5.19	6.43	0.44	Lower	5.25E-05	0.0400	0.11

- Un-paired two-tailed t test was used to calculate the *P*-value.
- Benjamini-Hochberg False Discovery Rate (FDR) correction was used to adjust the *P*-value.
- Significant changes in abundance levels are shown with an adjusted *P*-value <0.05
- AUC, Area under the curve

Supplementary Table 4: Significant inverse correlation between the identified miRNA and their potential target mRNAs.

mRNA	miRNA	Ebstein's anomaly Correlation	Ebstein's anomaly Correlation *P*-value	Controls Correlation	Controls Correlation *P*-value	Difference
CACUL1 (CDK2 associated cullin domain 1)	hsa-miR-23a-3p	0.48	0.0688	-0.39	0.1931	-0.87
CCNY (cyclin Y)	hsa-miR-30e-3p	0.63	0.0126	0.08	0.8065	-0.55
CDH1 (cadherin 1)	hsa-miR-340-3p	0.72	0.0033	-0.05	0.8775	-0.77
CDH1 (cadherin 1)	hsa-miR-326	0.46	0.0834	-0.18	0.5461	-0.65
CDH1 (cadherin 1)	hsa-miR-23b-3p	0.44	0.1043	-0.21	0.4924	-0.65
CDH1 (cadherin 1)	hsa-miR-142-3p	0.59	0.0232	0.05	0.8653	-0.54
CDH1 (cadherin 1)	hsa-miR-361-3p	0.61	0.0151	0.09	0.7665	-0.52
CDH1 (cadherin 1)	hsa-miR-505-3p	0.53	0.0438	0.01	0.9644	-0.52
CERS2 (ceramide synthase 2)	hsa-miR-340-3p	0.69	0.0044	-0.10	0.7370	-0.79
CHD4 (chromodomain helicase DNA binding protein 4)	hsa-miR-361-5p	-0.43	0.1070	0.32	0.2872	0.75
DGKD (diacylglycerol kinase delta)	hsa-miR-23a-3p	0.80	0.0004	-0.12	0.6932	-0.92
DGKD (diacylglycerol kinase delta)	hsa-miR-942-5p	0.54	0.0386	-0.30	0.3242	-0.84
DGKD (diacylglycerol kinase delta)	hsa-miR-30c-5p	0.48	0.0725	-0.35	0.2342	-0.83
DGKD (diacylglycerol kinase delta)	hsa-miR-30b-5p	0.86	0.0001	0.07	0.8201	-0.79
DGKD (diacylglycerol kinase delta)	hsa-miR-505-3p	0.59	0.0232	-0.15	0.6217	-0.74
DGKD (diacylglycerol kinase delta)	hsa-miR-361-3p	0.73	0.0021	0.11	0.7322	-0.62
DGKD (diacylglycerol kinase delta)	hsa-miR-199a-5p	0.76	0.0010	0.14	0.6536	-0.62
GPI (glucose-6-phosphate isomerase)	hsa-miR-339-5p	-0.70	0.0036	0.09	0.7818	0.79
THRAP3 (thyroid hormone receptor associated protein 3)	hsa-miR-23a-3p	0.63	0.0117	-0.33	0.2699	-0.96
THRAP3 (thyroid hormone receptor associated protein 3)	hsa-miR-199a-5p	0.58	0.0221	-0.31	0.2960	-0.90
THRAP3 (thyroid hormone receptor associated protein 3)	hsa-miR-340-3p	0.60	0.0195	0.04	0.8918	-0.56
ITGAM (integrin subunit alpha M)	hsa-miR-23b-3p	0.59	0.0201	-0.60	0.0290	-1.20
ITGAM (integrin subunit alpha M)	hsa-miR-23a-3p	0.39	0.1499	-0.48	0.0997	-0.87

ITGAM (integrin subunit alpha M)	hsa-miR-140-3p	0.52	0.0479	-0.22	0.4639	-0.74
KPNA6 (karyopherin subunit alpha 6)	hsa-miR-125a-5p	0.75	0.0019	-0.04	0.9062	-0.79
PTK2B (protein tyrosine kinase 2 beta)	hsa-miR-23a-3p	0.49	0.0620	-0.43	0.1456	-0.92
PTK2B (protein tyrosine kinase 2 beta)	hsa-miR-23b-3p	0.43	0.1125	-0.47	0.1019	-0.90
PTK2B (protein tyrosine kinase 2 beta)	hsa-miR-7-1-3p	0.36	0.1870	-0.53	0.0642	-0.89
PTK2B (protein tyrosine kinase 2 beta)	hsa-miR-378a-5p	0.37	0.1779	-0.49	0.0915	-0.85
PTK2B (protein tyrosine kinase 2 beta)	hsa-miR-340-3p	0.31	0.2535	-0.48	0.1013	-0.79
RNF130 (ring finger protein 130)	hsa-miR-140-3p	0.51	0.0536	-0.31	0.3046	-0.82
RNF130 (ring finger protein 130)	hsa-miR-23a-3p	0.55	0.0336	-0.16	0.6021	-0.71
RNF130 (ring finger protein 130)	hsa-miR-30b-5p	0.38	0.1596	-0.14	0.6415	-0.53
VPS26B (VPS26, retromer complex component B)	hsa-miR-30b-5p	0.55	0.0343	-0.63	0.0202	-1.18
VPS26B (VPS26, retromer complex component B)	hsa-miR-365a-3p	0.60	0.0213	-0.32	0.2872	-0.92
VPS26B (VPS26, retromer complex component B)	hsa-miR-340-3p	0.56	0.0336	-0.22	0.4704	-0.78
SCRN3 (secernin 3)	has-let-7b-5p	-0.28	0.3106	0.35	0.2378	0.63
